# Spatiotemporal characteristics of ground microtremor in advance of rockfalls

**DOI:** 10.1038/s41598-022-10611-3

**Published:** 2022-05-11

**Authors:** Yi-Rong Yang, Tzu-Tung Lee, Tai-Tien Wang

**Affiliations:** Department of Civil Engineering, National Taiwan University, Taipei, Taiwan

**Keywords:** Civil engineering, Natural hazards

## Abstract

Identifying cliffs that are prone to fall and providing a sufficient lead time for rockfall warning are crucial steps in disaster risk reduction and preventive maintenance work, especially that led by local governments. However, existing rockfall warning systems provide uncertain rockfall location forecasting and short warning times because the deformation and cracking of unstable slopes are not sufficiently detected by sensors before the rock collapses. Here, we introduce ground microtremor signals for early rockfall forecasting and demonstrate that microtremor characteristics can be used to detect unstable rock wedges on slopes, quantitatively describe the stability of slopes and lengthen the lead time for rockfall warning. We show that the change in the energy of ground microtremors can be an early precursor of rockfall and that the signal frequency decreases with slope instability. This finding indicates that ground microtremor signals are remarkably sensitive to slope stability. We conclude that microtremor characteristics can be used as an appropriate slope stability index for early rockfall warning systems and predicting the spatiotemporal characteristics of rockfall hazards. This early warning method has the advantages of providing a long lead time and on-demand monitoring, while increasing slope stability accessibility and prefailure location detectability.

## Introduction

Rock cliffs are usually unstable and may lead to catastrophic collapse that seriously threatens the construction of slope engineering and the safety of passengers in vehicles on the slope^1,2^. Only a few papers about rockfall prediction have been published, and no clear precursors before rock collapses have been detected^1,3–7^. Kinematic and deformation analysis methods, including the limit equilibrium method^8^, numerical simulation method^1,9–11^, fuzzy theory^12,13^ and acoustic emission^14^, are used in the stability evaluation of unstable rock slopes. These theoretical tools can analyse the spatiotemporal characteristics of rockfall hazards and are suitable for the assessment of the damage level of rock slopes but not for the accurate monitoring and early warning of a rockfall. Currently, the technologies used for landslides monitoring mainly include laser scanning methods, light detection and ranging systems (LiDAR), GPS receivers, geographical information systems (GISs), video image recognition systems and interferometric synthetic aperture radar (InSAR)^4,15–19^. The InSAR and ground-based radar techniques have been developed to identify potential failure in rock slopes^20^. These remote image analysis techniques can provide forecasting rockfall locations and warning times of minutes^21^. Although another landslide early warning system based on rainfall monitoring can lead to a longer warning time of days^22^, this system can be applied only over specific regions and for rainfall-induced landslides.

With the advancement of electronic technology, some researchers have started to monitor unstable rock slopes and indicated that the landslide seismic response and vibration signal monitoring index can offer a foundation for the early warning of rock collapse^9,23,24^. The ground vibrates before collapse due to crack nucleation and propagation in the unstable rock. The vibration amplitude and fundamental vibration frequency can record failure precursors in unstable rocks^25^. When a rock slope becomes dangerous, the measured seismic response exhibits strong directional amplification^26,27^. Compared to displacement analysis, these results show that vibration analysis signal characteristics can be used to improve early warning approaches of rock collapse^2,3^. However, seismic monitoring has temporal and spatial limitations because it can record signals after a seismic event in only a seismic zone. For monitoring and early warning of rockfall hazards, choosing an appropriate technique for rockfall hazard assessment is therefore necessary.

Microtremors are small-scale ambient vibrations of the ground caused by natural phenomena and have been applied to study sedimentary thickness, evaluate local site effects and locate weathered bedrock depths and landslide areas^28–30^. In this study, we introduce ground microtremor signals for early rockfall forecasting and conduct in situ and laboratory experiments to study the relationship between microtremor characteristics and slope stability. We then compare the effects of three early warning detection systems based on microtremor, displacement and crack generation characteristics. Our analysis demonstrates that microtremor characteristics provide the earliest precursor of rockfall and are remarkably sensitive to slope stability. To this end, we apply a microtremor-based rockfall warning method on a mountain highway in central Taiwan to identify cliff areas that are prone to fall and provide appropriate warnings according to the estimated slope stability.

## Methods

To study the microtremor characteristics of mountain slopes, an in-situ experiment was conducted with accelerometer sensors on Highway No. 8 (also known as the Tai-8 highway, Central Cross-Island Highway) in central Taiwan. A series of laboratory tests were conducted to evaluate the relationship between the FS and microtremor characteristics of overhanging rock and establish a rock slope stability extraction method.

### Microtremor monitoring on a mountain slope

**Figure 1:**
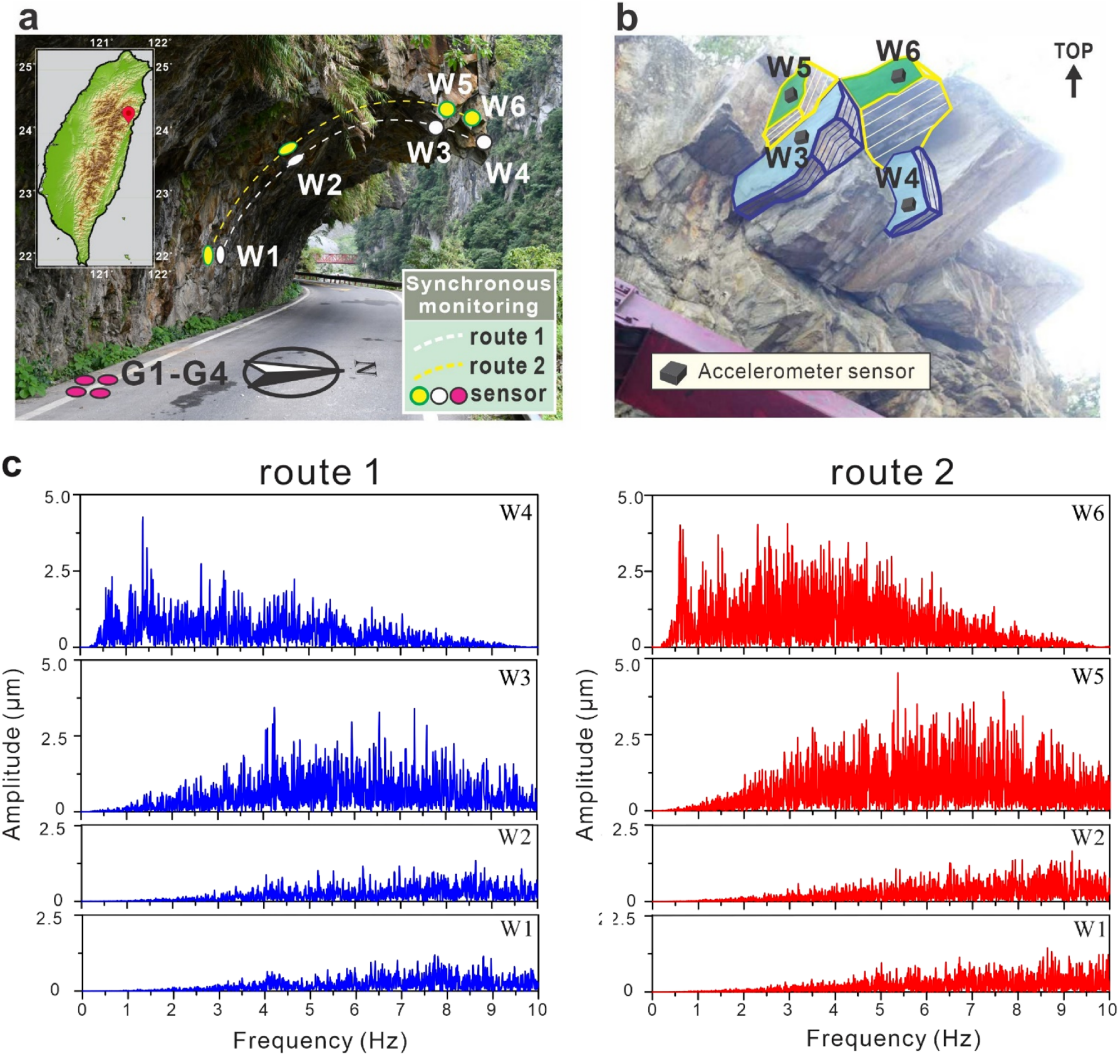
Microtremor monitoring of rock slope neighbouring mountain highways in central Taiwan. (**a**) Site photograph of the field experiment on the overhanging slope along the old Tai-8 highway and locations of the measurement points. Overview map showing the study region within Taiwan. (**b**) Photograph of the overhanging rock wedges monitored by the accelerometer sensors on the slope. (**c**) Fast Fourier transform results of the microtremor signals in the vertical direction of measurement points on route 1 (left) and on route 2 (right).

An in-situ experiment was conducted on a rerouted section near Taroko, Hualien of the Tai-8 highway (Fig. 1). The Tai-8 highway runs through an extremely unstable and rugged region of the Central Mountain Range in Taiwan. Heavy rain from monsoons and typhoons and considerable shocks by earthquakes often cause substantial rockfall damage to highways and other transportation infrastructures^31^. Four MEMS triaxis accelerometer sensors SDI 2422H low voltage 5 V DC with a sensitivity up to 2000 mV/g were set to monitor the microtremor signals synchronously. In order to record the microtremor signal of hard gneiss wedge on slopes, high-sensitivity accelerometer sensors were mounted directly on target rock for microtremor measurement in low-to-medium frequency. The microtremors at the ground level (G1–G4) of a rerouted section of the old Tai-8 highway, neighbouring lateral slope walls (W1 and W2) and overhanging cliffs (W3, W5, W4 and W6) were recorded by triaxial accelerometer sensors (Fig. 1a,b). All the sensors were installed along two synchronous monitoring routes with a distance of 2–8 m (Fig. 1a). Microtremors were measured for 180 s at least three times at each synchronous monitoring route for selecting record data free from transient conflicts. The moving average filter was employed on microtremor signals to reduce random noise. The record data had been digitally band-pass-filtered in the 0.2–10 Hz frequency range in the time domain to remove high frequency disturbances. For understanding the characteristics of microtremor signal, the Hilbert–Huang transform (HHT) and the fast Fourier transform (FFT) algorithm were used to convert the complex microtremor signal from original time domain into a representation in the frequency domain. The former decomposes microtremor signal into the instantaneous frequency of each intrinsic mode function, and generates a frequency-time distribution of signal amplitude. The latter processor assists in analysing microtremor signals of certain frequency band in detail and eliminating environmental noise. After that, the Gaussian frequency-domain and moving average filter windows were applied to suppress the spectral leakage and smooth the discontinuity at the edges of the FFT slices.

### Physical rockfall experiment

**Figure 2:**
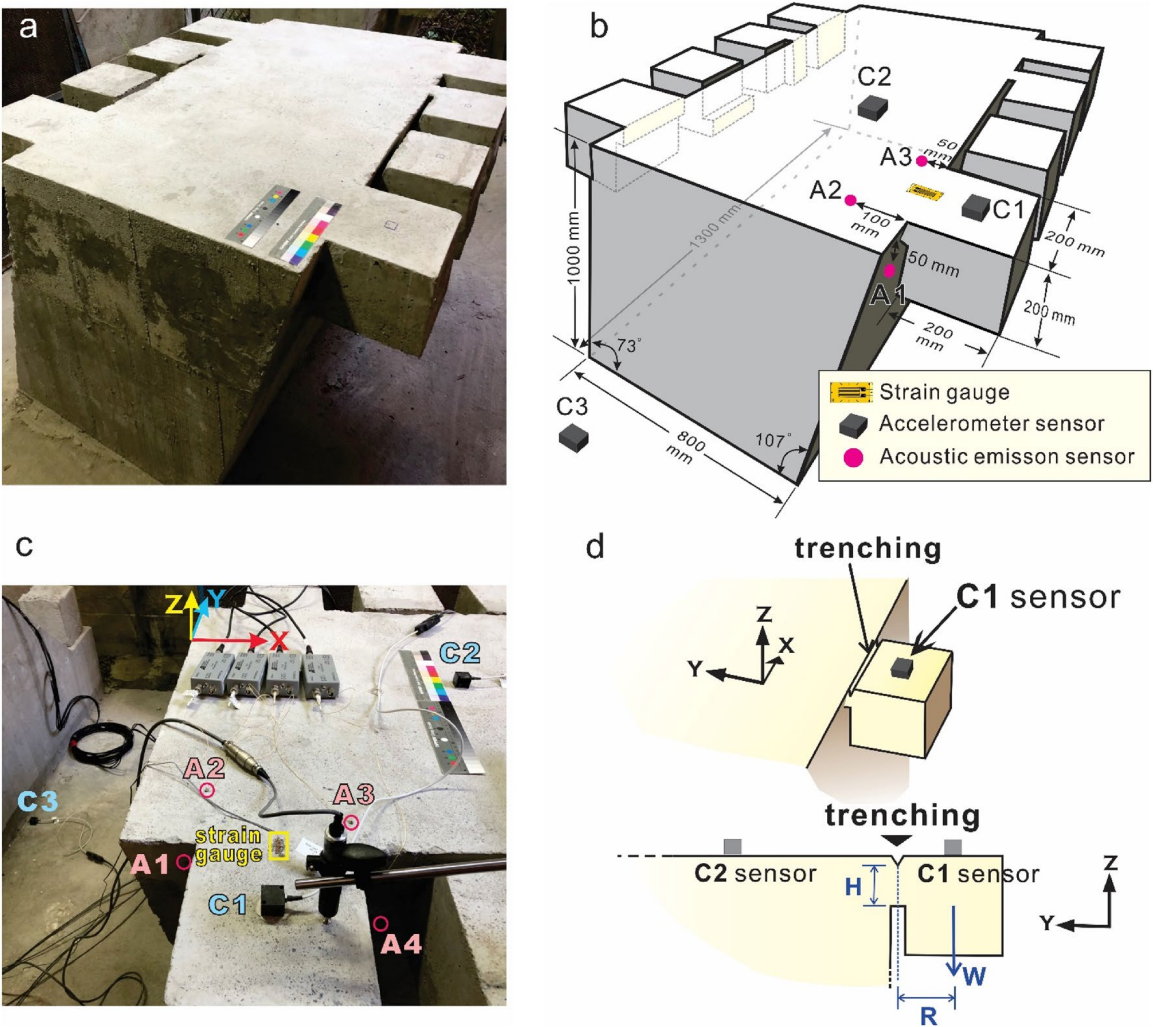
Physical rockfall experiment. (**a**) Photograph of concrete specimen. (**b**) Three-dimensional sketch of the physical rockfall experiment. (**c**) Locations of the accelerometers (C1–C3), strain gauge, linear variable differential transformer and acoustic emission sensors (A1–A4). (**d**) Oblique and side views of the artificial trenches between the main block and overhanging block, which are designed to simulate the bedrock and overhanging rock wedge, respectively.

We executed a series of physical rockfall experiments. A main block with an overhanging block made of plain concrete, with a density of 2.3 × 10^3^ kg/cm^3^ and a uniaxial compressive strength of 21 MPa, was prepared to model the bedrock slope and overhanging rock wedge. Figure 2 shows the physical model and measuring system. A strain gauge, three accelerometer sensors and four acoustic emission sensors were set on the specimen to monitor the displacement, microtremor signal and crack development, respectively.

To study the relationship between the slope stability and the microtremor characteristics, the junction between the overhanging block and main block was cut down gradually (Fig. 2d). To quantitatively describe the rock stability on the slope, we added progressive loading on the overhanging block. The microtremor characteristics of the rock were obtained by an accelerometer sensor, and the FSs were obtained through cantilever beam moment calculation.

## Results

### Microtremor monitoring of rock slope

In the in-situ experiment, W1 and W2 mounted on slope walls reflect similar ground microtremor patterns as those of the signals observed at the ground level (G1–G4), which shows that the fundamental frequency in this area is in the range of 6–10 Hz and that the amplitudes are below 1.3 µm (Fig. 1). Along the routes, the amplitudes of the microtremors recorded at two protruding rock wedges, W3 and W5, became larger, and the frequency ranges decrease slightly. The outermost cliffs, W4 and W6, located at the end of routes, show higher amplitudes of up to 4 µm and relatively low dominant frequencies of 2–6 Hz. The result indicates that the microtremor characteristics may be highly sensitive to the stability of rock wedges and can indicate the location of unstable rock wedges on slopes before they occur. To evaluate the relationship between microtremor characteristics and slope stability, a series of laboratory tests were conducted.

### Microtremor characteristics

**Figure 3:**
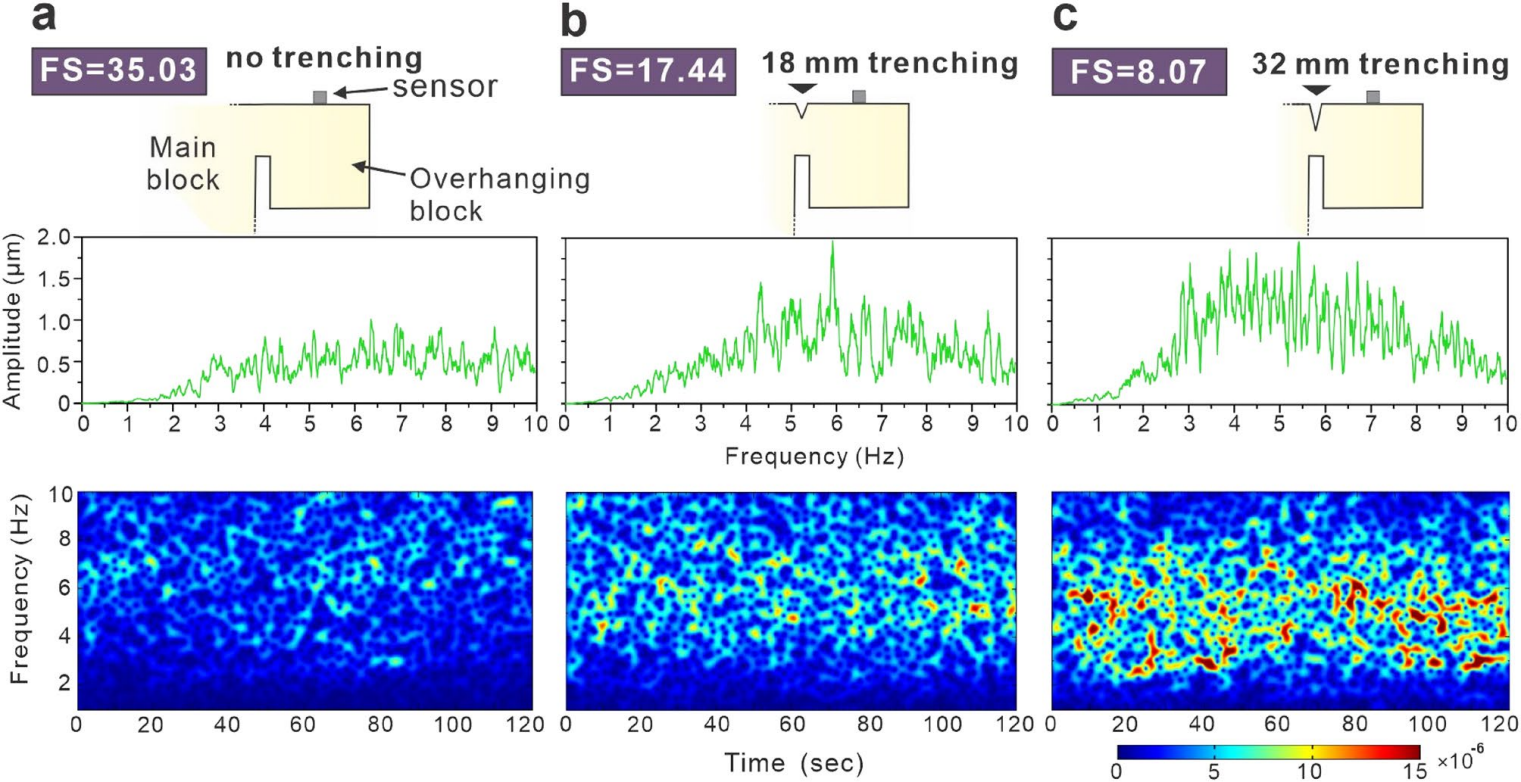
Schematic diagram of sensor installation, microtremor signals in the vertical direction of the overhanging block and fast Fourier transform of microtremor signals. (**a**) Microtremor signals of the overhanging block without an upper trench. (**b**) Microtremor signals of the overhanging block including an 18 mm-deep upper trench. (**c**) Microtremor signals of the overhanging block including a 32 mm-deep upper trench.

In the laboratory tests, a main block with an overhanging block made of plain concrete was prepared to model the bedrock slope and overhanging rock wedge (Fig. 2). The concrete has a uniaxial compressive strength of 21 MPa and a tensile strength ($\sigma_{strength}^{t}$) of 3.73 MPa. Three different contact areas between these blocks were considered. First, the overhanging block was supported by a 200 × 63 mm junction to the main block without an upper trench (Fig. 3a). Second, an 18 mm-deep trench was cut artificially at the top of the junction (Fig. 3b). Third, a 32 mm-depth upper trench was considered (Fig. 3c). The moment at the junction part *M*_*rx*_ that is generated by the weight of overhanging block (*W*) and the vertical component of applying loading (*F*_*z*_) has a magnitude of *R*(*W* + *F*_*z*_), where *R* is the distance between the connection point of main block and the overhanging block. Since the plane concrete is brittle, the deflection of the junction part is small and each cross-section of the part is perpendicular to its neutral axis. Thus, the maximum tensile stress $\sigma_{max}^{t}$ occurs at the top of the junction part and can be calculated using Euler–Bernoulli beam theory, written as

1 $$\sigma_{max}^{t} = \frac{{M_{rx} }}{{I_{x} }} \cdot \frac{H}{2},$$

where *I*_*x*_ is the second moment of area of the beam's cross-section with respect to the x axis, and *H* is the height of the junction part. The factor of safety (FS) of an overhanging block is then calculated as

2 $$FS = \frac{{\sigma_{strength}^{t} }}{{\sigma_{max}^{t} }}.$$

The calculated FSs for these three conditions are 35.03, 17.44 and 8.07. The narrow contact surface with a lower FS reflects a relatively unstable rock slope. The microtremor signals in the vertical component of the overhanging blocks and the FFT patterns are shown in Fig. 3. This result suggests that as the overhanging block became unstable, the amplitude of the vertical microtremor signal was relatively large, and the energy in the time domain was concentrated in the lower frequency band of the 2–6 Hz range (Fig. 3c). Current early warning methods can warn of a rockfall when the FS is close to 1.0. The results of laboratory tests indicate that ground microtremor signals are remarkably sensitive to rock slope stability, even when the FS of the rock slope is greater than 8. This finding demonstrates that ground microtremors are an early precursor of rockfall and have the potential to determine a rock slope stability index.

### Stability related analytical frequency

**Figure 4:**
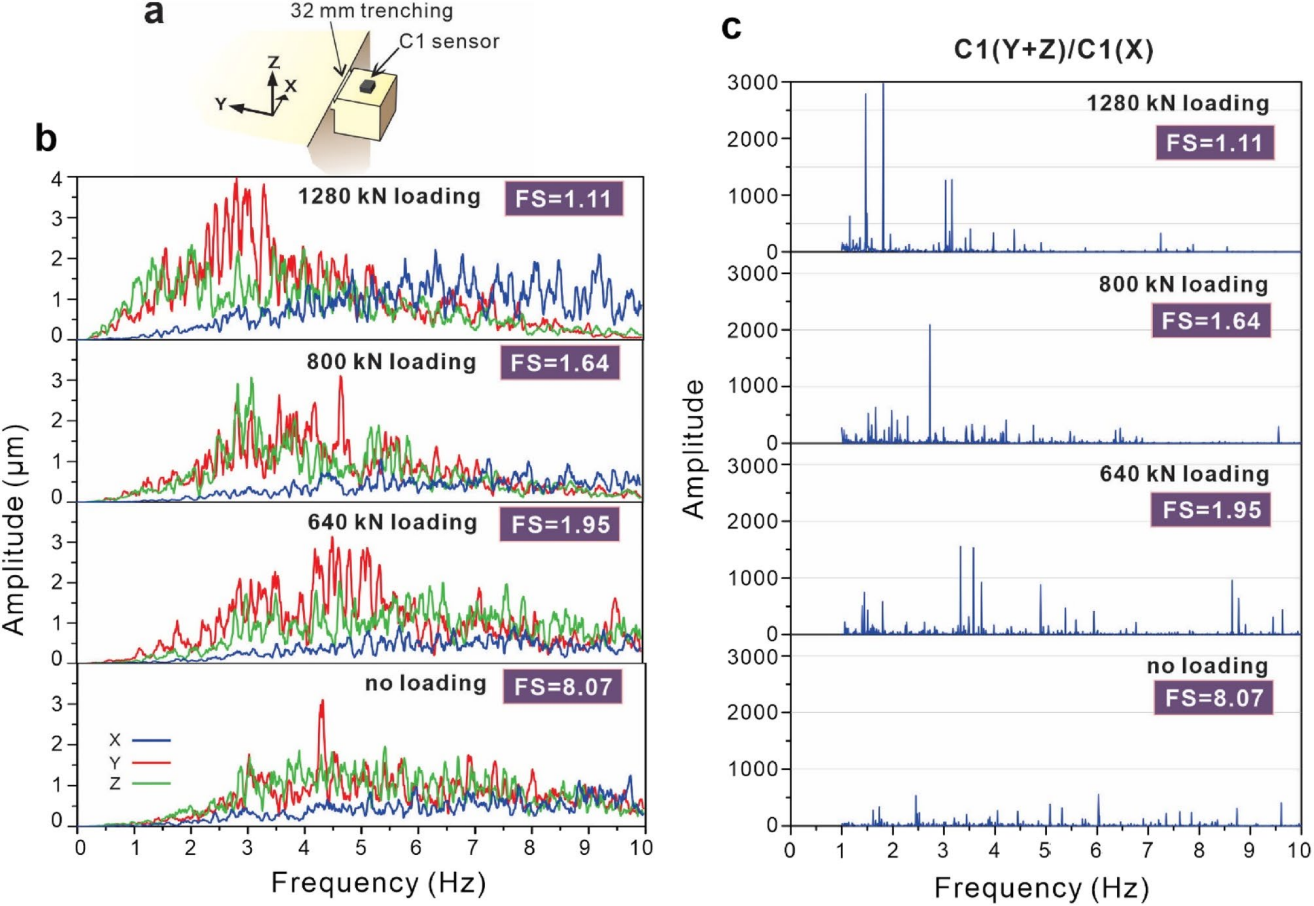
Measured and feature extracted microtremor signals of the overhanging block under progressive loading. (**a**) Schematic diagram of the overhanging specimen including a 32 mm-deep upper trench. (**b**) Measured microtremor signals, decomposed into three components, of the overhanging block under progressive loading. (**c**) Feature extracted microtremor signals. The result from the sum of the horizontal components towards the main block (y-axis) and the vertical component (z-axis) together, divided by the horizontal tangential component, towards the x-axis.

The weight and size of unstable rock cliffs vary, which may interfere with the identification of unstable rock slope derived from microtremor characteristics. To extract the rock stability-related signal information, we designed a laboratory test to reduce the signal information caused by rock mass (Fig. 2). To simulate varied rock wedge masses, we added progressive loading on the overhanging block supported by the junction with a 32 mm-depth upper trench (Fig. 4a). The block finally fell when the load was increased to 1440 kN. We assumed that the FS was 1.0 when the overhanging block fell and determine the tensile strength ($\sigma_{strength}^{t}$) of the concrete according to Eq. (1). Then the FSs in all statuses of the overhanging specimen can be calculated using Eq. (2). Figure 4b shows the three components of the microtremor signals of the overhanging block. Before the block fell, the microtremor amplitude increased and the microtremor frequency decreased as the designed loads increased. However, the weight/load of the block mass also contributed similar characteristics to the block vibration. To amplify the microtremor characteristics and reduce the mass-induced vibration effects, we added the signals in the horizontal component towards the main block (y-axis) and the vertical component (z-axis), i.e., $Am_{hor} = \sqrt {\left( {Am_{Y} } \right)^{2} + \left( {Am_{Z} } \right)^{2} }$ and then divided them by the horizontal tangential component, towards the x-axis *Am*_*x*_. The microtremor signal in the horizontal tangential component, towards the x-axis, is the only signal component that does not reflect the block stability but has the same mass component as the others. Therefore, we chose this component as a divisor to remove the mass-induced vibration effects and extract the features of rock slope stability from the microtremor signals. The feature extraction results are presented in Fig. 4c. With increasing rock mass/load, the amplitude ratio increased sharply from 500 to 3000, and the analytical frequency shifted from 6 to 1.5 Hz.

## Discussion

**Figure 5:**
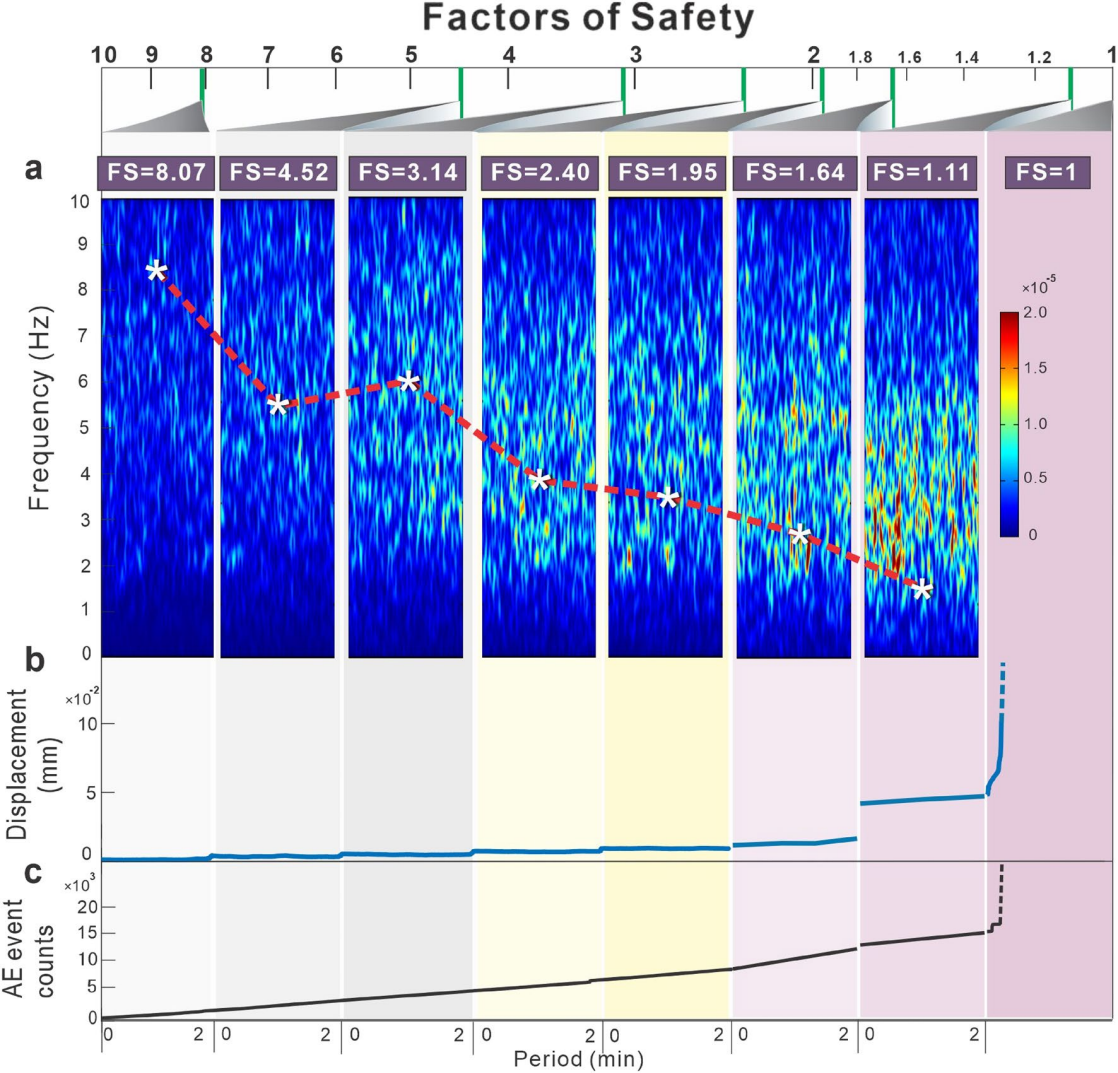
Comparison between microtremor frequency, block displacement, factor of safety and crack generation under progressive loading. (**a**) Power spectrum of the vertical component of the measured microtremor signals in the overhanging block. Red dotted lines and white asterisks show the analytical frequency using the extraction method. (**b**) Displacements of unstable blocks under progressive loading. (**c**) Crack generation indexed using the acoustic emission event counts of unstable blocks under progressive loading.

Figure 5a shows the effect of this rock slope stability extraction method. With the FS of rock slope, power spectrum of the original microtremor signals in the vertical component from the overhanging block varies remarkably. Red dotted lines and white asterisks show the analytical frequency using the extraction method. The analytical frequency clearly decreases compared to the original signals as the block becomes unstable, especially when the FS is lower than 2.0. This indicates that the rock slope stability extraction method enhances the sensitivity of ground microtremor signals to slope stability and can help to more accurately detect the location of unstable rock slopes in the field and provide more lead time for landslide remediation works.

### Rockfall predictability

To compare the effects of current early warning detection systems based on slope displacement, crack generation and microtremor characteristics, we used the sensors to monitor the unstable overhanging block (Fig. 5). For a microtremor-based warning system, the energy of the microtremor signal provides the very first precursor of rockfall by concentrating at a low frequency when the FS decreases (Fig. 5a). Both the original microtremor frequency and extracted feature frequency decrease noticeably as the rock becomes unstable, until the block falls. For the displacement-based warning system, the displacement of the unstable block does not show significant precursors before a rockfall (Fig. 5b). The displacement increases sharply only when the FS drops to 1.11. The corresponding displacement magnitude, smaller than a millimetre, is too small for current remote imaging sensors to detect in the field. The crack-based warning system shows that the acoustic emission event count increases consistently throughout the experimental process (Fig. 5c). There is no significant crack amount as an index of precursors for early rockfall warning because rock always failures with various crack amount. Compared with displacement- and crack-based warning systems, the microtremor-based warning method exhibits three main points corresponding to rockfall warnings. One advantage of this system is that the microtremor-based warning method provides the earliest lead time. Second, the stability of the slope can be quantitatively described with the FS from the microtremor frequency of the slope. Three, the microtremor monitoring setup, including accelerometer sensors, is simple, fast, portable and cost-effective. Ground microtremors can be monitored at any time, unlike with the seismic signal-based warning method, which requires monitoring after seismic events. To summarize the above results, the microtremor-based warning method is the most appropriate technique for rockfall hazard assessment.

Theoretically, FS ≤ 1 indicates failure stage, when 1 < FS ≤ critical FS the slope is considered potential failure conditions and FS > critical FS denotes stable conditions. Based on slope investigations, the critical FS is between 1.07 and 1.25^32–36^. From Fig. 5, the critical FS corresponds to approximate 1.5 Hz extracted feature frequency and the microtremor signal energy concentrates at a narrow band of 1–6 Hz. To apply the microtremor-based warning method on rockfall hazard assessment, the first rockfall warning is suggested to be issued when the extracted feature frequency of microtremor signal of a slope drops to the risk identification index, 1.5 Hz, or the signal energy concentrates at a low frequency of 1–6 Hz. After that, numerical analysis method^9–11,37,38^ can be introduced to combine the climate and geological environment for investigating the rock landslide deformation and proposing the slope mitigation plan.

Microtremor characteristics can assist system managers of government to formulate an efficient multiple rockfall warning system. If the microtremor characteristics of a cliff or rock wedge show that the signal energy is concentrated at a low frequency or extracted feature frequency of microtremor signal drops to 1.5 Hz, a highway administrative unit should decide the warning type, a prerockfall watch should commence, and a rockfall warning system should be utilized according to the corresponding FSs before the rockfall. Taking the mountain slope of the old Tai-8 highway as an example (Fig. 1), both the original microtremor signals and feature extracted signals we analysed at W1 and W2 show that the analytical frequencies are larger than 6 Hz. According to the frequency FS chart (Fig. 5a), the corresponding FSs are greater than 4.5, which means that there is no rockfall risk. The corresponding FSs of W3 and W5 are approximately 4.0, relatively stable according to safety factor classifications^32–36^. The results of the sensors at the outermost cliff areas, W4 and W6, show low frequencies of less than 2 Hz, and the corresponding FSs are lower than 1.64. The analytical frequency at W4 is nearly 1 Hz. Thus, it is suggested that the highway administrative unit continues monitoring the microtremor signal of W6 and analyses the slope stability or immediately remove the potential failure rock mass at W4 to prevent a rockfall from occurring.

## Conclusions

Establishing an efficient rockfall warning system plays an important role in rockfall prevention. Without a clearly detectible precursor before rock collapse, the predictability of current early-warning systems for rockfall has been limited. Our findings show that the ground microtremor signal is remarkably sensitive to slope stability. We therefore propose that the microtremor characteristics can provide a very early precursor of rockfall to detect unstable rock on slopes early and quantize the stability of slopes. Such microtremor characteristics may be applied to field investigations for slope reinforcement construction. Extending the lead time of microtremor-based warnings will reduce the rockfall hazard risk posed to the safety of nearby people, construction sites, rockfall-prone roads and some cultural heritages.

This study highlights the potential of microtremor signal to identify precursors to rockfall demonstrated by simple physical experiments. For the promotion of this microtremor analysis technique, it will be a future research to understand the influence of lithological characters, joint sets, slope roughness, etc., on microtremor signal of rock wedge on slopes. Even though the accelerometer sensor is sensitive enough for well detecting the low-amplitude microtremor signal, it still holds limitations of short-distance monitoring and single-station measurement, mainly because that the accelerometer sensor is mounted directly on the target rock wedge to collect data. Thus, to improve the measurement device for extending the application of microtremor-based warning method to the widely environments of rockfall prediction is also a key issue in the future.
